# Supplementary material for: Elicitation of Broadly Neutralizing Antibodies against B.1.1.7, B.1.351, and B.1.617.1 SARS-CoV-2 Variants by Three Prototype Strain-Derived Recombinant Protein Vaccines
Source: Viruses. 2021 Jul 22;13(8):1421. doi: 10.3390/v13081421 (PMC8402859; doi:10.3390/v13081421)
Supplement: Supplementary file 1 [file viruses-13-01421-s001.zip › viruses-1289052-supplementary.pdf]

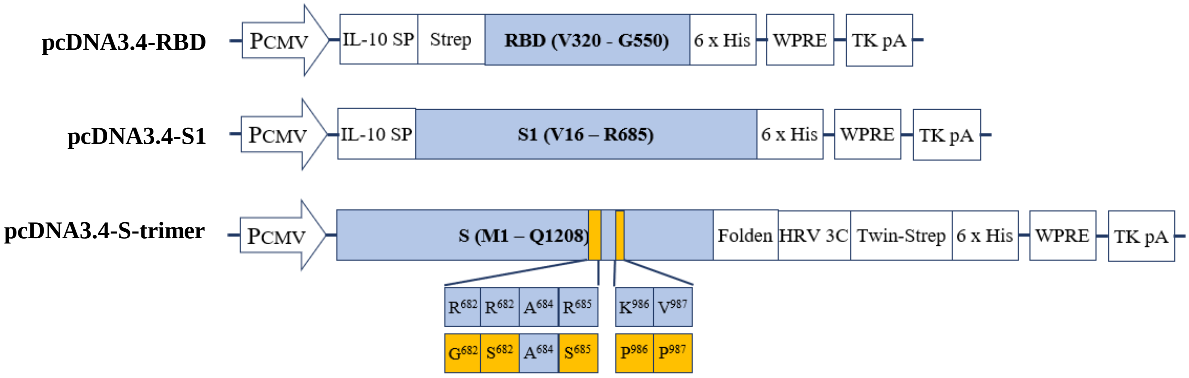

Supplementary Figure S1. Schematic diagrams of recombinant expression vectors used in this study. P_{CMV}, human cytomegalovirus promoter; IL-10 SP, human interleukin 10 signal peptide; Strep, Strep-tag II; WPRE, woodchuck hepatitis virus posttranscriptional regulatory element; TK pA, herpes simplex virus thymidine kinase polyadenylation signal; Folden, T4 fibritin trimerization motif; HRV 3C site, human rhinovirus 3C protease cleavage site; Twin-Strep, Twin-Strep-tag. Note that recombinant S-trimer protein was stabilized by the "RRAR" to "GSAS" substitution to disrupt the furin S1/S2 cleavage site and the double proline mutation at the junction of heptad repeat 1 (HR1) and central helix (CH).

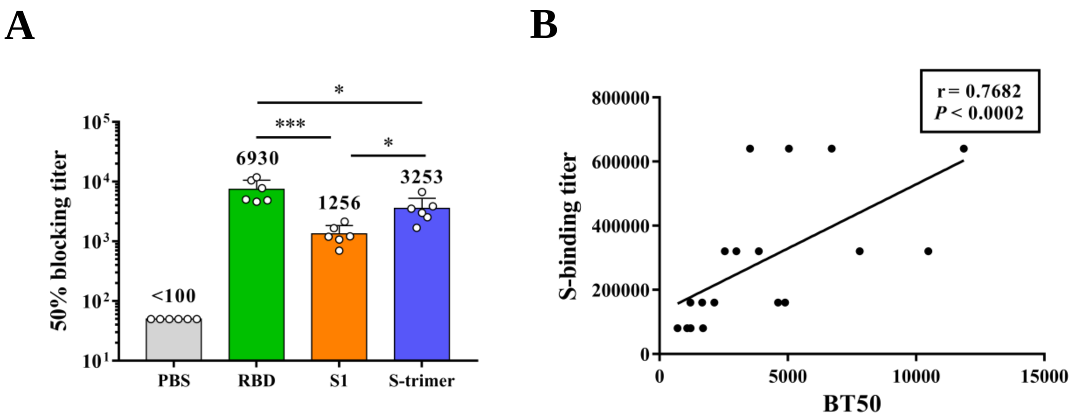

Supplementary Figure S2. Receptor binding-inhibition ability of the immune sera. **(A)** The hACE2/S-trimer binding-inhibition titers of the week-18 anti-sera were determined by ELISA. Statistical significance was determined by Student's t-test and is indicated as follows: * $p < 0.05$, ** $p < 0.01$, *** $p < 0.001$. Error bars represent SD. **(B)** Correlation between hACE2/S-trimer binding-blockade titers (BT50) and S-binding titers of the week-18 anti-sera. Spearman rank-correlation test was used.

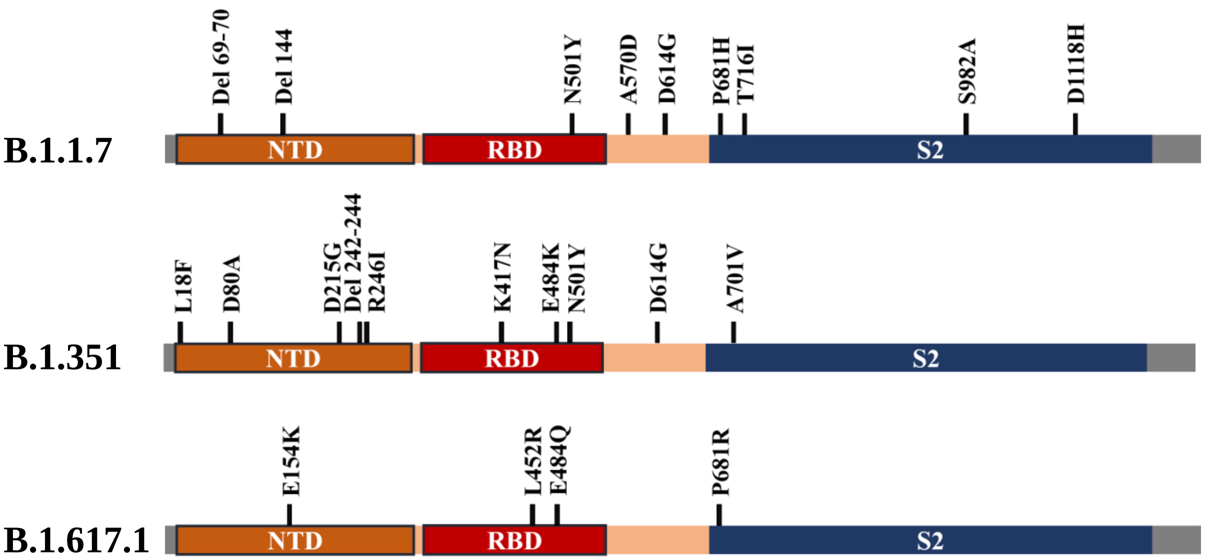

Supplementary Figure S3. Schematic diagrams of the spikes of SARS-CoV-2 variants B.1.1.7, B.1.351 and B.1.617.1. Mutations are shown at the top of each diagram.
